# Analysis of Treatment and Prognosis of 863 Patients with Spinal Tuberculosis in Guizhou Province

**DOI:** 10.1155/2018/3265735

**Published:** 2018-09-23

**Authors:** Guangru Cao, JingCheng Rao, Yuqiang Cai, Chong Wang, Wenbo Liao, Taiyong Chen, Jianpu Qin, Hao Yuan, Peng Wang

**Affiliations:** Department of Spine Surgery, Affiliated Hospital of Zunyi Medical College, Zunyi 563000 Guizhou, China

## Abstract

The objective of this study was to investigate the treatment and prognosis of patients with spinal tuberculosis in Guizhou province. A total of 863 patients with spinal tuberculosis admitted to our hospital from 2006 to 2017 were included in this study. All patients underwent standardized quadruple antituberculosis treatment. Eighty patients were lost to follow-up due to a change of their contact information or noncompliance. A total of 783 patients completed the follow-up. The average follow-up period was 20.33 ± 8.77 months (range: 6 to 38 months). Among these patients, 145 patients underwent conservative treatment, while 638 patients underwent surgical treatment. All patients in the surgery group were treated with lesion removal, bone graft fusion, and internal fixation. Preoperative and postoperative standard quadruple antituberculosis treatment was administered. The clinical efficacy was evaluated according to erythrocyte sedimentation rate (ESR), c-reactive protein (CRP), visual analogue scale (VAS), Cobb angle correction, neurological functional recovery, and interbody fusion with bone graft and tuberculosis outcome. A total of 608 patients achieved clinical cure. The symptoms, physical signs, blood tests and imaging findings were improved in 143 patients. Twenty patients showed refractory clinical symptoms, and 12 patients had local tuberculosis recurrence. Conservative and surgical treatments are the mainstream treatments for spinal tuberculosis. According to the patients' individual conditions, individualized treatments should be used to achieve good efficacy. Standardized antituberculosis treatment should be applied over the course of spinal tuberculosis.

## 1. Introduction

China is a region with a higher incidence of tuberculosis and ranks second worldwide in the total number of patients with tuberculosis. Tuberculosis is a serious threat to public health in China. The latest research shows that 10.4 million people fell ill with tuberculosis and there were approximately 6.3 million new tuberculosis cases in the world in 2016 [1]. In 2012, Erdem H reported that TB ranks second, just after HIV infection, among infectious causes of mortality [2]. Guizhou is a relatively poor and economically undeveloped province with a large rural population and poor living conditions in its mountainous areas. There are a large number of people suffering from pulmonary tuberculosis and extrapulmonary tuberculosis [3]. Osteoarticular tuberculosis is a common secondary extrapulmonary tuberculosis, of which spinal tuberculosis accounts for 50%. Spinal tuberculosis is common in young adults, responsible for kyphosis and paraplegia, associated with a high disability rate and difficult to treat. Spinal tuberculosis mainly infects weight-bearing joints with frequent movement. Thoracic (40% to 50%), lumbar (35% to 45%), and cervical (10%) vertebrae are common sites for infection [4]. In clinical practice, tuberculosis patients with definite surgical indications should receive standardized antituberculosis therapy and then undergo lesion removal, bone graft fusion and internal fixation [5].

## 2. Materials and Methods

### 2.1. Eligibility Criteria

*Diagnostic Criteria for Spinal Tuberculosis*. (1) Previous history of pulmonary tuberculosis or other extrapulmonary tuberculosis: the patient has the chest and back pain, emaciation, symptoms of low fever, neurological dysfunction, the experience antituberculosis treatment is effective; (2) imaging studies: magnetic resonance imaging (MRI) and computed tomography (CT) consistent with spinal tuberculosis; (3) erythrocyte sedimentation rate (ESR), c-reactive protein (CRP) increased to varying degrees; (4) microbiologic evidence included at least one of the following: isolation of M. tuberculosis in blood, bone, deep soft tissues or (paravertebral, epidural, or psoas) abscess specimens; positive microscopy for acid-fast bacilli from bone, deep soft tissue or (paravertebral, epidural, or psoas) abscess (Ziehl–Neelsen staining); M. tuberculosis culture positive.

*Rehabilitation Criteria for Spinal Tuberculosis*. (1) Patients with chest and back pain, emaciation, and symptoms of low fever disappeared; (2) CT, MRI, and other imaging data, no recurrence of tuberculosis was found, bone union in the bone graft area; (3) At the final follow-up of the patient, no complications occurred; (4) erythrocyte sedimentation rate (ESR), c-reactive protein (CRP) decreased to normal. Final follow-up visual analogue scale (VAS), Cobb angle, and neurological dysfunction were restored to varying degrees.

*Inclusion Criteria for Patients in the Conservative Treatment Group*. (1) Patients with complete stability of the spine and negative imaging findings; (2) patients with the initial onset of mild clinical symptoms but without deformities and neurological dysfunction; (3) patients with less than 30° thoracic or thoracolumbar kyphosis and normal cervical or lumbar physiological curves.

*Inclusion Criteria for Patients in the Surgical Treatment Group*. (1) Patients with a large cold abscess or gravitation abscess; (2) progressive or severe neurological deficit; (3) presentation of large sequestrum and cavity within the lesion; (4) intractable sinus tract or refractory infection; (5) obviously unstable spine, severe or progressive spinal kyphosis.

*Exclusion Criteria for Patients in the Surgical Treatment Group*. (1) Patients complicated with severe heart and lung dysfunction who cannot tolerate surgery; (2) pregnant and lactating women; (3) patients complicated with active tuberculosis.

### 2.2. Management

#### 2.2.1. Treatment

Quadruple antituberculosis agents (isoniazid 0.3 g/d, rifampicin 0.45 g/d, ethambutol 0.75 g/d, and pyrazinamide 0.75 g/d) were administered to patients with a stable spine, negative imaging findings, initial onset, and mild clinical symptoms. For patients without neurological dysfunction or with mild neurological dysfunction who met the criteria for inclusion in the surgical group, the same 2 weeks quadruple antituberculosis agents were routinely administered preoperatively. In patients with progressive aggravating neurological dysfunction, surgical treatment should be performed immediately after 5-7 days of standardized antituberculosis treatment to relieve the compression of the lesion tissue on the spinal cord or the cauda equina and promote the recovery of neurological function. Radiography, CT scan, and MRI were required before surgery to locate the lesion and determine the appropriate surgical approach. All patients should take 18 months of antituberculosis drugs.

#### 2.2.2. Surgical Procedures

**Figure 1:**
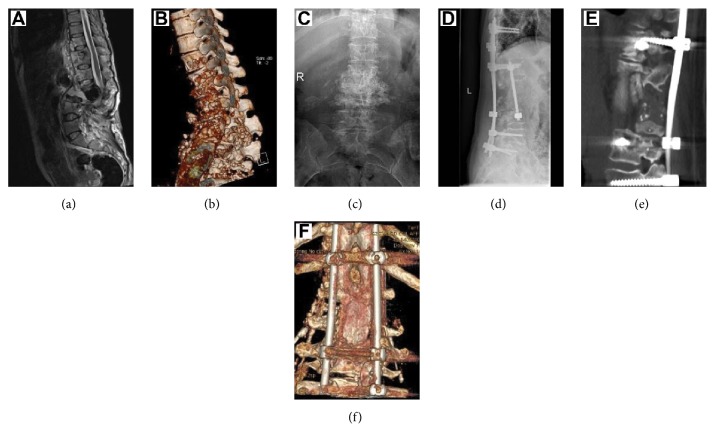
(a)-(c) Preoperative imaging data of a 40-year-old female showing T10 to L4 vertebral destruction, paravertebral soft tissue shadow widening, and a tuberculosis lesion protruding into her spinal canal and compressing her dural sac. (d)-(f) Postoperative imaging study showing that the tuberculosis lesion has been completely removed and that the position of the internal fixation device is satisfactory. Postoperative bone bridge formation is visible in the diseased vertebral body.

**Figure 2:**
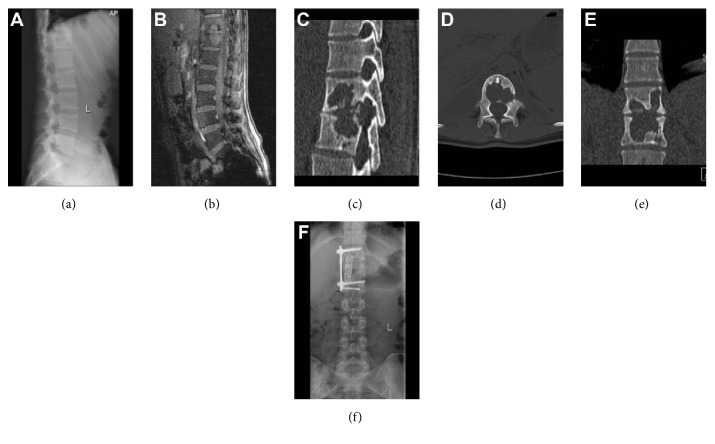
(a) Radiograph of a 25-year-old woman showing T11 and T12 vertebral destruction. (b) Sagittal MRI view showing T11 and T12 vertebral destruction with paravertebral and intra-spinal abscesses. (c) Sagittal CT scan view showing T11 and T12 vertebral destruction. (d) Axial CT scan view showing T11 vertebral destruction. (e) Coronal CT scan view showing T11 and T12 vertebral destruction. (f) The lateral-anterior approach was used to perform lesion removal of T11 and T12 vertebral tuberculosis, placement of intervertebral titanium mesh, bone grafting, fusion, and internal fixation. A postoperative radiograph shows satisfactory positioning of the internal fixation device and complete removal of the tuberculosis lesion.

We have taken lateral approach, anterior approach, posterior approach, and anterior approach combined with posterior approach, as shown in Figures 1 and 2.

#### 2.2.3. Postoperative Management

Lesion tissue excised during surgery was sent for pathological examination. Intraoperative aspiration of pus was tested for mycobacterium tuberculosis. On the third postoperative day, ESR, CRP, and haemoglobin levels were tested again. Antibiotics were routinely used for 3-5 days after surgery to prevent infection. The patients continued standardized antituberculosis treatment for 12-18 months. The drainage tube for the surgical wound was removed when the drainage amount was less than 50 ml per day. Radiography, CT scan, and MRI were repeated every 3 months after surgery to observe bone graft fusion and signs of local recurrence of spinal tuberculosis. Liver and kidney functions were tested concurrently, in order to identify the side effects of the medications.

### 2.3. Observation Parameters

Clinical efficacy was evaluated during follow-up according to ESR, CRP, VAS, Cobb angle correction, neurological functional recovery, interbody fusion with bone graft, and tuberculosis outcome.

## 3. Results

### 3.1. General Data

A total of 863 patients with spinal tuberculosis who were admitted to our hospital from 2006 to 2017 were included in this study. All patients underwent standardized quadruple antituberculosis treatment. Eighty patients were lost to follow-up due to a change in their contact information or noncompliance. A total of 783 patients completed the follow-up. The average follow-up period was 20.33 ± 8.77 months (range: 6 to 38 months). There were 357 males and 426 females, of whom 145 were on conservative treatment and 638 received surgical treatment. Among them, 9 were diagnosed with cervical tuberculosis, 293 with thoracic tuberculosis, 126 with thoracolumbar tuberculosis, 314 with lumbar tuberculosis, and 41 with sacral tuberculosis. The clinical manifestations included local chronic pain and limited mobility, paravertebral abscess or gravitation abscess, neurological dysfunction, and spinal deformity. Of 393 patients with neurological dysfunction, 69 patients had degenerative neurological dysfunction. According to the Frankel classification (Grade A: completely paralyzed; Grade B: preserved sensation only and no motor function; Grade C: preserved sensory function and nonfunctional motor function; Grade D: preserved sensory function and functional motor function; Grade E: normal motor and sensory function), there were 17 patients classified as grade A, 52 as grade B, 138 as grade C, 186 as grade D, and 390 as grade E. The details are shown in Table 1. The average preoperative Cobb angle of the vertebrae was 17.68 ± 4.38°, and the average preoperative pain visual analogue scale (VAS), erythrocyte sedimentation rate (ESR) and C-reactive protein (CRP) levels were 6.90 ± 2.00, 44.65 ± 16.30 mm/h and 53.34 ± 38.21 mg/L, respectively. Imaging findings included the following: vertebral destruction or collapse, narrowed or eliminated intervertebral space, paravertebral abscess or gravitation abscess formation, compression of abscess, and necrotic tissue to the spinal cord and the cauda equina. A total of 252 patients were complicated with paravertebral abscess, 41 patients with intraspinal abscess, 261 patients with both paravertebral and intraspinal abscess, and 44 patients with psoas abscess. In our analysis of 783 bacteriologically/histopathologically confirmed cases of spinal tuberculosis, 677 (86.5%) patients had a typical MRI picture. This study was approved by the Hospital Ethics Committee and all patients in the surgery group met the inclusion criteria and signed the informed consent form.

### 3.2. Prognosis of Patients in the Conservative Treatment Group

Of 145 patients undergoing conservative treatment, 107 were cured clinically, 29 achieved relief of clinical symptoms, 5 had no response to the treatment, and 4 had recurrent tuberculosis. In 31 patients with neurological impairment, the Frankel grade at the final follow-up was improved from Grade D to Grade E after standardized quadruple antituberculosis treatment. Of the 9 patients with poor efficacy, 7 had found multidrug-resistant M. tuberculosis by drug sensitivity test.

### 3.3. Prognosis of Patients in the Surgical Treatment Group

**Figure 3:**
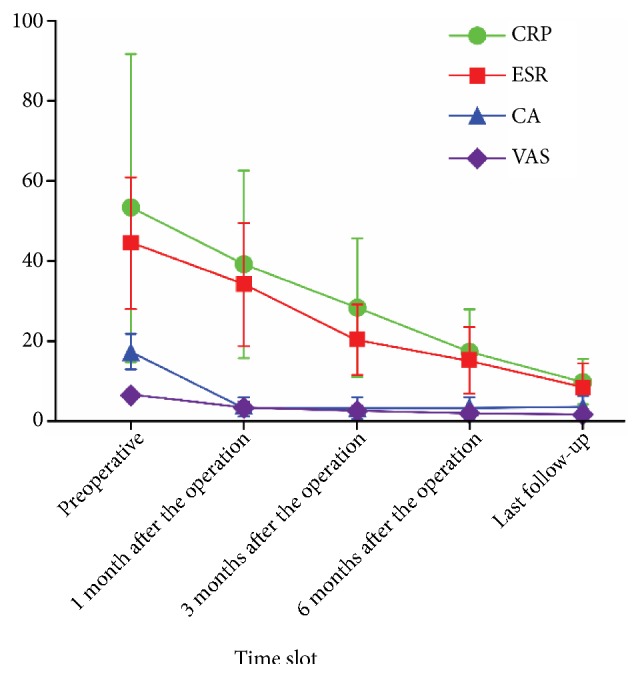
Trends of the related parameters of the patients undergoing surgery.

Of 638 patients undergoing surgical treatment, 501 were cured clinically, 114 achieved relief of clinical symptoms, 15 had no response to the treatment, and 8 had recurrent tuberculosis. At the final follow-up, the VAS score significantly improved from 6.90 ± 2.00 preoperatively to 1.99 ± 0.81 postoperatively (P < 0.05). As shown in Table 2, the Cobb angle significantly decreased from 17.68 ± 4.38° preoperatively to 3.99 ± 2.34° postoperatively (P < 0.05). The postoperative neurological function improved to a certain extent in 336 patients. The details are shown in Table 3 and Figure 3. Of the 23 patients with poor efficacy, 19 had found multidrug-resistant M. tuberculosis by drug sensitivity test.

## 4. Discussion

Spinal tuberculosis is a serious threat to public health. Spinal tuberculosis is common in patients who are in poor health and have a history of primary or secondary pulmonary syndrome TB [6]. Percival Pott [7] first described spinal tuberculosis in 1782. Guizhou Province has a high incidence of tuberculosis because of its relatively economical underdevelopment, large rural population, inconvenient transportation system, and low public awareness of spinal tuberculosis [8]. Unlike the study reported by Wang et al. [9], the vast majority of patients in this study lived in rural areas, had low incomes, and could not afford treatment. Spinal tuberculosis, the most common bone and joint tuberculosis, is also a common and frequently encountered disease in Guizhou.

To date, the treatment of spinal tuberculosis includes conservative and surgical treatments. Chemotherapy is considered a sufficient treatment, as determined by the British Medical Research Council Working Party on Spinal Tuberculosis [10]. Of 145 patients undergoing conservative treatment in this study, 107 were cured clinically, 29 achieved relief of clinical symptoms, 5 had no response to the treatment, and 4 had recurrent tuberculosis. Studies have shown [11] that 69% of patients undergoing conservative treatment can achieve satisfactory results. Of 638 patients undergoing surgical treatment in this study, 501 were cured clinically, 114 achieved relief of clinical symptoms, 15 had no response to the treatment, and 8 had recurrent tuberculosis. Causes for the poor prognosis of patients may include the following: low income, poor nutritional status, other concurrent systemic diseases, poor compliance with taking antituberculosis drugs, drug-resistant tuberculosis mycobacteria, compromised stability of the spine which affected the bony fusion between diseased vertebrae, and incomplete intraoperative removal of lesions. The World Health Organization's annual report for 2017 indicates that the incidence of tuberculosis in developing and backward countries is significantly higher than that in developed countries [1]. Spinal tuberculosis is a chronic wasting disease. Most patients have symptoms such as weight loss, anemia, and hypoproteinemia. Other concurrent systemic diseases make the body more in a high-consumption state and cannot create a good environment for the rehabilitation of patients with spinal tuberculosis. The nutritional status of the system is crucial to the outcome of the disease. Poor compliance with taking antituberculosis drugs can lead to incomplete treatment and even drug-resistant M. tuberculosis. Drug-resistant Mycobacterium tuberculosis can affect the osseous fusion between the diseased vertebrae, destroy the stability of the spine, and cause spinal deformity and neurological dysfunction. The spread of drug-resistant M. tuberculosis has exacerbated the global epidemic of tuberculosis, which poses a serious threat to spinal tuberculosis. Incomplete intraoperative removal of lesions can lead to recurrent spinal tuberculosis, requiring a second surgical cleanup.

Spinal instability is an indication for surgical treatment. In patients with tuberculous kyphosis, surgical treatment is more effective than conservative treatment [12]. Lateral, anterior, posterior, and combined anterior with posterior approaches, as well as spine osteotomies, have been described as procedures to correct spinal sequences and restore sagittal balance [13, 14]. Standardized antituberculosis drug therapy should be applied over the course of spinal tuberculosis [15, 16]. Each procedure has its advantages and disadvantages. According to the specific conditions of different patients, the appropriate surgical procedure should be selected to achieve individualized treatment.

With the prevalence of multidrug-resistant mycobacterium tuberculosis, some patients are resistant to first-line anti-tuberculosis agents, including isoniazid and rifampicin. However, conventional drug-sensitive tests require 2-3 months to obtain results and cannot be counted on for adjusting the antituberculosis treatment regimen in a timely manner. Therefore, the delayed treatment will accelerate the progression of neurological impairment and kyphosis and negatively affect the prognosis of patients [17–19]. Compared with drug-sensitive mycobacterium tuberculosis, drug-resistant mycobacterium tuberculosis is a current challenge in the medical community [20]. Studies have shown that the incidence of spinal tuberculosis combined with pulmonary tuberculosis ranges from 26% to 32% [21–23]. In this study, 249 patients (31.8%) had pulmonary tuberculosis and spinal tuberculosis concurrently.

Consistent with other studies [24], the vast majority of spinal tuberculosis in this study occurred in the thoracolumbar spine. In our study, the most common complications were abscess (78.7%) and neurological deficit (50.2%), which were higher than those of Erdem H et al. [25]. Jain AK [26] believes that neurologic complications are the most dreaded complications of spinal tuberculosis, and neurological deficits are unfavourable factors that affect the prognosis of spinal tuberculosis. Erdem H [25] also believes that the presence of neurologic deficits is one of the predictors of unfavourable outcome. The findings from this study are in line with this belief. The postoperative prognosis of patients with spinal paralysis caused by the compression of spinal tuberculosis tissue and patients with compromised spinal stability due to spinal tuberculosis is poorer than that of patients without corresponding changes [25, 27]. Some researchers [28] believe that a great number of vertebral bodies affected by tuberculosis is associated with a poor postoperative prognosis. This result is consistent with the data from the current study.

**Figure 4:**
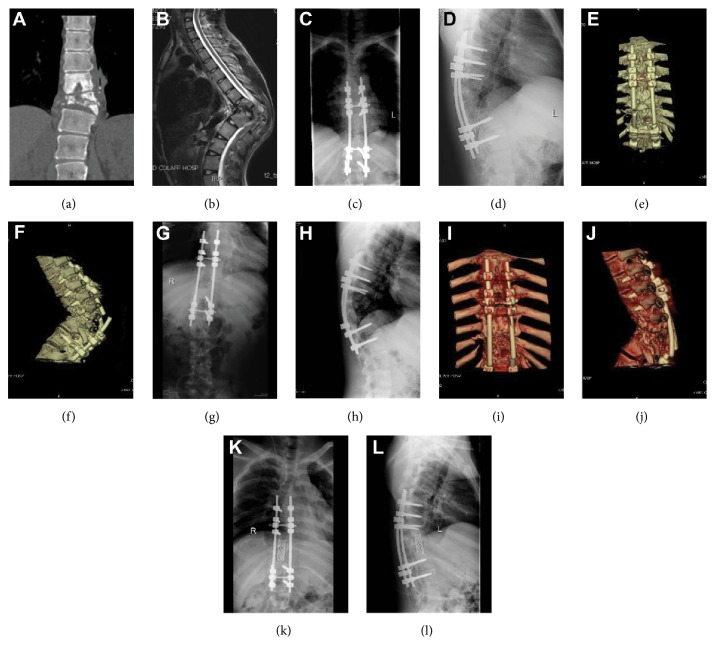
(a)-(b) Preoperative imaging study of an 18-year-old male showing thoracic vertebral tuberculosis and thoracic kyphosis. (c)-(d) The posterior approach was used for surgery, and postoperative imaging data show satisfactory positioning of the internal fixation device. (e)-(f) Three-dimensional reconstruction of CT scan images showing breakage of the internal fixation device after surgery. (g)-(h) The posterior approach was used for the initial repair surgery. The anteroposterior and lateral radiographic views show satisfactory positioning of the internal fixation device after surgery. (i)-(j) Three-dimensional reconstruction of CT images showing breakage of the internal fixation device after the initial repair surgery. (k)-(l) The combined anterior and posterior approach was used for the secondary repair surgery. Anteroposterior and lateral radiographic views show satisfactory positioning of the internal fixation device. The lesion has been completely removed.

After treatment, the majority of patients in this study achieved relief or improvement of symptoms, including chest and back pain, results of imaging studies, ESR, CRP, and neurological function to varying degrees. However, Figure 4 shows broken screws and plates in a patient undergoing surgery via the posterior approach due to the failure to completely remove the lesion during surgery, poor support of implanted bone powder, and lack of a large iliac bone implant. During reoperation, the combined approaches were used to repair the spine. The anterior approach was used to completely remove the lesion and to implant the autologous iliac bone. The posterior approach was used to place internal fixation implants. Neither broken screws and plates nor spinal tuberculosis recurrence were reported during follow-up. To this end, the combined anterior with posterior approach is the optimal option to treat patients with complex spinal tuberculosis. This conclusion is in line with the study of Vamvanij V [29].

## 5. Conclusions

In our analysis of 783 bacteriologically/histopathologically confirmed cases of spinal tuberculosis, 677 (86.5%) patients had a typical MRI picture, which was comparable with the study conducted by A Sharma in New Delhi who found 264 (84.62%) patients had a typical MRI picture [30]. Conservative medical treatment in patients without deformities and neurological dysfunction resulted in clinically successful therapeutic responses in 93.8% of the patients in our study, which is close to Kotil K's research [31]. Erdem H [25] studied 314 patients with spinal tuberculosis from 35 centres showed that lumbar vertebrae was the most commonly involved sites and that the vast majority of patients had paravertebral and intraspinal abscesses, which is in line with our study. In our study, 81.5% of patients required surgical intervention. The rate of surgery was higher than in Erdem H's research. This study has some limitations because it was a retrospective study with a limited data. To treat spinal tuberculosis is still a challenge clinically. The Chinese Government and the entire medical industry must increase their investment in the prevention and treatment of tuberculosis, especially in rural and underdeveloped regions.

## Figures and Tables

**Table 1:** General patient information.

	Conservative treatment (145)	Surgical treatment (638)	Statistical analysis
Gender (male/female)	66/79	291/347	Z=-10,549, P=0.121
Age (years)	47.32±19.33	44.65±16.30	t=1.716, P=0.086
Lesion section			
Cervical vertebrae	2	7	
Thoracic vertebrae	54	239	
Thoracolumbar	23	103	
Lumbar	58	256	
Sacral vertebrae	8	33	
Frankel classification			
A	0	17	
B	0	52	
C	0	138	
D	31	155	
E	114	276	
Complicated abscess site			
Paravertebral	47	205	
Intraspinal	8	33	
Paravertebral and intraspinal	48	213	
Psoas	8	36	
Iliac fossa	3	15	
None	31	136	
Preoperative VAS score	6.91±2.15	6.90±2.00	t=0.040, P=0.968
Operation approach			
Lateral approach		381	
Anterior approach		32	
Posterior approach		18	
Combined anterior approach with posterior approach		207	

**Table 2:** Parameter changes over the disease course in patients undergoing surgery.

	CRP	ESR	Cobb	VAS score
Before surgery	53.34±38.21	44.65±16.30	17.68±4.38	6.90±2.00
1 month after surgery	39.35±23.30*∗*	34.25±15.19*∗*	3.83±2.41*∗*^#^	3.90±1.41*∗*
3 months after surgery	28.60±17.15*∗*	20.67±8.73*∗*	3.84±2.37*∗*^#^	2.93±1.40*∗*
6 months after surgery	17.73±10.53*∗*	15.56±8.29*∗*	3.90±2.35*∗*^#^	2.50±1.11*∗*
Final follow-up	10.23±5.71*∗*	8.85±5.92*∗*	3.99±2.34*∗*^#^	1.99±0.81*∗*

Note: *∗* versus data before surgery, p<0.05; ^#^ between the time points, p>0.05.

**Table 3:** Changes in Frankel classification before surgery and at the last follow-up.

Before surgery		Final follow-up				
		A	B	C	D	E
A	17	3	2	4	6	2
B	52			15	20	17
C	138				37	101
D	155				23	132
E	276					276

## Data Availability

The data used to support the findings of this study are available from the corresponding author upon request.
